# Fatal Disruption of a Left Atrial Myxoma Associated with Trauma

**DOI:** 10.1155/2012/486309

**Published:** 2012-03-26

**Authors:** Anthony Iacco, Nazneen Billimoria, Greg Howells

**Affiliations:** Department of Surgery, Beaumont Health System, 3601 W. Thirteen Mile Road, Royal Oak, MI 48073, USA

## Abstract

Cardiac myxomas are benign tumors composed of sparse stellate cells in an extensive mucoid stroma. The surface of these tumors is often friable and gelatinous. Their intracardiac location makes embolization a constant threat. We report a patient who had diffuse systemic embolization of a left atrial myxoma coincident with a low-velocity frontal motor vehicle crash.

## 1. Introduction

Cardiac myxomas are the most common type of primary cardiac tumor. Most patients present with nonspecific symptoms. However, patients can present with obstructive or embolic signs. Herein, we report the case of a 26-year-old male with diffuse systemic embolization of a left atrial myxoma following a motor vehicle crash.

## 2. Case Report

 The patient is a 26-year-old man brought to our Emergency Center from the scene of a frontal crash in which the patient's vehicle impacted a wall at 30 mph. The patient was unrestrained and air bags did not deploy.

 Upon EMS arrival the patient was unresponsive and had a witnessed seizure. En route to the hospital the patient received Valium, Narcan, and IV fluids without a change in his mental status.

 Upon arrival in the Emergency Center the patient's airway was intact, his respiratory rate was 47 breaths per minute, blood pressure in the left brachial artery was 62/55 mmHg, and heart rate was 132 beats per minute. The lower extremities were mottled from the mid-thigh level with ischemic changes in both feet and absent pedal pulses. The left radial pulse and both femoral pulses were weak, but the right radial pulse was normal. Pupils were fixed and dilated bilaterally and Glasgow Coma Scale score was 7. No evidence of trauma was present on physical exam of the head, chest, abdomen, or extremities. Blood pressure was retaken in the right brachial artery and found to be 116/76 mmHg.

 The patient was intubated and a right femoral arterial line was placed and confirmed the blood pressure of 60/50 mmHg at the femoral level.

**Figure 1:**
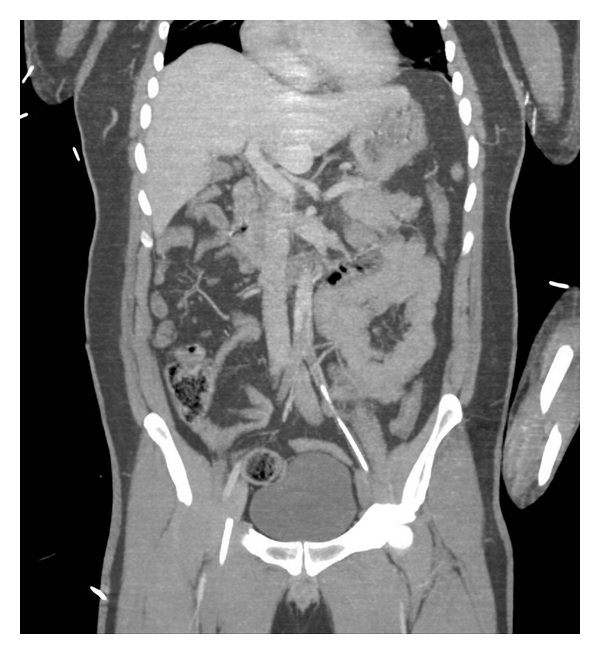
Abdominal CT showed multiple splenic and renal infarcts and a thrombus at the aortic bifurcation extending into the inferior mesenteric artery and into both common iliac arteries.

A presumptive diagnosis of blunt aortic injury with pseudocoarctation syndrome was made and the patient was rapidly transported for a head, chest, abdomen, and pelvis computerized tomography (CT) scan. The head CT was normal. The chest study showed a large left atrial filling defect attached to the atrial septum and an abrupt cutoff of the left axillary artery. Abdominal CT showed multiple splenic and renal infarcts and a thrombus at the aortic bifurcation extending into the inferior mesenteric artery and into both common iliac arteries (Figure 1).

**Figure 2:**
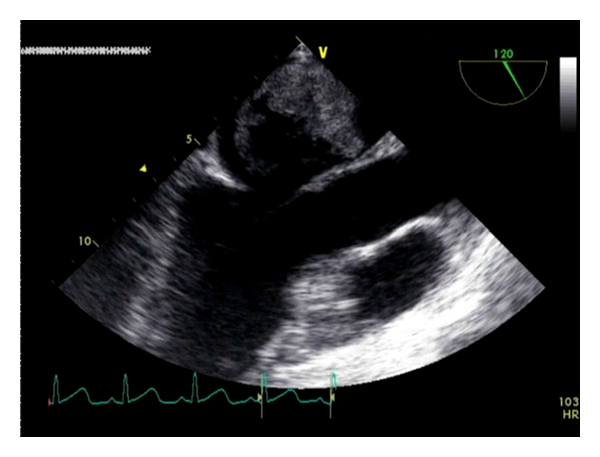
Transesophageal echocardiography showing left atrial myxoma.

 The patient was returned to the trauma bay where he underwent transthoracic and transesophageal echocardiography (Figure 2). These revealed a 3-4 cm left atrial pedunculated mass. The mass was spherical with a large part missing, reminiscent of an apple with a bite out of it. The diagnosis of left atrial myxoma versus sarcoma with extensive embolization was made.

 At surgery the patient underwent an axillary artery embolectomy with reestablishment of left upper extremity circulation. Bilateral femoral embolectomies were done. Though clot was adequately removed from the femoral popliteal systems, retrograde aortoiliac embolectomies were inadequate, and laparotomy with transaortic antegrade embolectomies as required to reestablish lower extremity flow. Pathology from femoral, brachial, and aortic embolectomies revealed organizing thrombus consistent with emboli from atrial myxoma. The small bowel and colon were viable at laparotomy. Cardiovascular surgery evaluated the patient preoperatively and the decision was made not to proceed with removal of the atrial mass until after the patient was more stable and showed signs of neurological activity.

**Figure 3:**
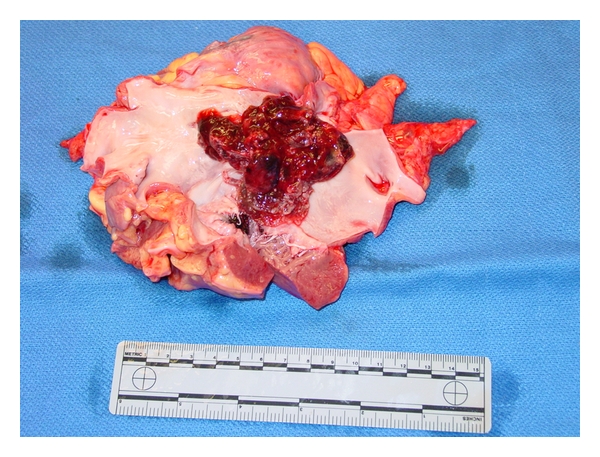
Left atrium opened showing myxoma.

 Postoperatively the patient lost gag and corneal reflexes. Repeat head CT scan showed diffuse cerebral edema with impending herniation. Supportive care was withdrawn at the family's request and the patient expired on postoperative day one. Autopsy confirmed a 7 × 4 × 1 cm left atrial myxoma (Figure 3). Microscopic exam revealed a left atrial tumor with stellate or globular myxoma cells.

## 3. Discussion

 Myxomas account for 30–50% of all primary cardiac tumors [1]. It is a benign tumor histologically; however, metastasis may occur from embolization from the primary tumor. Although they can occur in all four cardiac chambers, 83% arise in the left atrium [2]. They are gelatinous, friable tumors with a tendency to embolize as in our case. However, they can also produce congestive heart failure by mechanical obstruction of the mitral valve. Pulmonary embolization can occur with right atrial tumors [3].

Clinical manifestations depend upon the location and size of the tumor. The symptoms caused by myxomas fall within one of three groups which comprise the classic triad. They are obstructive cardiac signs, embolic signs, and constitutional/systemic signs.

Obstructive cardiac findings are the most common symptoms within the triad. They include dizziness, dyspnea, cough, pulmonary edema, and congestive heart failure. These symptoms occur when the tumor causes mechanical obstruction of the mitral valve. Abnormal electrocardiogram findings, frequently showing left atrial enlargement, are present in about two-thirds of patients. Embolic signs can present as either systemic or pulmonary depending on the tumor location and patency of the foramen ovale. Cardiac myxomas are the most common primary cardiac tumors to produce emboli that can travel to any organ or tissue [1]. Constitutional/systemic signs are nonspecific and include fever, weight loss, fatigue, myalgias, arthralgias, muscle weakness, and Raynaud's syndrome [4, 5].

According to most large case series reports of patients with cardiac myxomas, obstructive cardiovascular symptoms were present in 50–94% of patients, systemic embolization was present in 20–29% of patients, and constitutional symptoms were present in 11–34% of patients [3, 6–8]. Left atrial and left ventricular myxomas most commonly embolize to cerebral, retinal, and coronary arteries [9, 10]. It is also known that myxomas can embolize to the abdominal aorta including its many branches [11]. Embolism of myxomatous tissue can be spontaneous, but can also be associated with infection of bacteria or fungus, with consequent septic emboli and possible endocarditis [12, 13].

Echocardiography is the most common method of diagnosis. Transesophageal echocardiography is the recommended modality for initial assessment. Magnetic resonance imaging is principally used for preoperative purposes, although cardiac CT is also used [1].

 Removal of the tumor is indicated to prevent these sequelae. Mortality rates for the elective procedure are in the 3–5% range [6, 7]. Other postoperative complications include cardiac arrhythmias and conduction abnormalities. Recurrence rates range from 2–5% and can occur anywhere from 3 months to 14 years postoperatively [3]. The site can be local or extracardiac such as brain, lung, skeletal muscle, bone, kidney, gastrointestinal tract, skin, and other soft tissues. A very rare complication is the formation of cerebral aneurysms secondary to embolic tumor fragments which can be fatal [10].

There is little published on the association of atrial myxoma and rupture in the setting of trauma [14]. We have speculated that fracture and embolization of the tumor occurred on frontal impact in this unrestrained patient. Alternatively, it is possible the tumor fractured spontaneously and contributed to the crash in the first place. The multiple embolectomies were facilitated by two surgical teams. We assume he also had multiple emboli in the cerebral circulation, which were not amenable to embolectomy and were ultimately responsible for his demise.
